# Evaluation of cardiovascular risk factors in patients with familial hypercholesterolemia from the North-Eastern area of Romania

**DOI:** 10.1186/s12944-020-01428-y

**Published:** 2021-01-11

**Authors:** Cristiana-Elena Vlad, Liliana Foia, Laura Florea, Irina-Iuliana Costache, Andreea Covic, Roxana Popescu, Delia Reurean-Pintilei, Adrian Covic

**Affiliations:** 1 Department of Nephrology-Internal Medicine, “Dr. C. I. Parhon” Clinical Hospital, Iasi, Romania; 2 Grigore T. Popa University of Medicine and Pharmacy, Iasi, Romania; Universitatii street, 700115 Iasi, Romania; 3 The Academy of Romanian Scientists, Bucharest, Romania

**Keywords:** Familial hypercholesterolemia, Cardiovascular risk factors, Atherosclerotic cardiovascular disease, Low density lipoprotein cholesterol, Ankle-brachial index, Carotid intima-media thickness

## Abstract

**Background:**

Familial hypercholesterolemia(FH) is one of the most frequent and important monogenic cholesterol pathologies. Traditional and non-traditional cardiovascular risk factors increase the prevalence of atherosclerotic cardiovascular disease(ASCVD) in this population. The aims of the study were: (a) to identify FH patients in the North-Eastern part of Romania and to analyze demographic, clinical and paraclinical data (b) to evaluate the risk of new cardiovascular events at follow-up in FH patients stratified by lipid-lowering agents.

**Methods:**

This first prospective study in the North-Eastern part of Romania was carried out between October 2017 and October 2019; out of 980 patients with dyslipidemia evaluated with the Dutch Lipid Network(DLCN) and Simon Broome(SM) scores, 61 patients with DLCN score above 3 and possible/probable FH(SM score) were included.

**Results:**

Nine hundred-eighty patients were examined and 61 (6.2%) were received the clinical diagnosis of FH. The mean age was 48.5±12.5 years, with more female patients than male patients (63.9% versus 36%). Hypertension was the main cardiovascular risk factor for both genders, followed by physical inactivity and obesity for the female group and active smoking for the male group. The measured DLCN score recorded: “possible” FH identified in 39.4%, “probable” FH in 45.9% and “definite” FH in 14.7%. The effective lipid-lowering drugs used were statin alone and statin in association with fenofibrate, which improved both the lipid profile values and the subclinical atherosclerosis markers (ankle-brachial index, carotid intima-media thickness and high-sensitivity C-reactive protein). New ASCVDs that emerged during the study were most commonly represented by coronary heart disease and stroke. At the same time, the new cardiovascular events were delayed in patients receiving the lipid-lowering drugs, without significant differences between them.

**Conclusions:**

In patients with suspected FH, the lipid-lowering agents during the follow-up period delayed the new cardiovascular events, yet failed to reach the goals proposed by the guidelines.

## Background

Atherosclerosis is a complex multifactorial disorder, consisting of chronic inflammatory response which causes plaque formation in the intima and media of medium and large arteries [1]. Atherosclerosis can affect the vessels throughout the life of an individual, with several risk factors being significant contributors to the atherosclerotic plaques formation: dyslipidemia, diabetes mellitus, and high blood pressure [1, 2].

The genetic cornerstone of atherosclerotic cardiovascular disease (ASCVD) includes both common deoxyribonucleic acid (DNA) variants (leading to polygenic susceptibility) and rare DNA versions that cause monogenic disorders. The typical monogenic disease is familial hypercholesterolemia (FH) [3]. FH is an autosomal-dominant pathology, identified in all races and ethnic groups and determined by mutations of the *low density lipoprotein receptor* (*LDLR), apolipoprotein B (APOB), proprotein convertase subtilisin/kexin type 9 (PCSK9)* genes, and it is considered a cause of premature coronary atherosclerotic disease [4, 5]. In Romania, the prevalence of FH records 1/213 for the heterozygous form [6] compared to other Caucasian populations with 1/500 individuals and to 1/1,000,000 for the homozygous form [7]. There are three available criteria for the clinical diagnosis of FH: the Simon Broome criteria, the Dutch Lipid Clinic Network Criteria (DLCNC) and the MedPed criteria [8, 9].

Familial and personal history of hypertension, coronary heart disease (CHD), stroke, chronic kidney disease, smoking, eating habits, alcohol consumption, a sedentary lifestyle and nontraditional risk factors represented by the ankle-brachial index (ABI) and high-sensitivity C-reactive protein (hsCRP) were significantly associated with cardiovascular morbidity and mortality in FH patients [10, 11]. Atherosclerotic plaques and the increase of mean cIMT were associated with a significantly expanded CHD risk [12, 13].

The management of cardiovascular risk factors is important for FH patients who associate cardiovascular atherosclerotic pathology, while statin therapy combined with ezetimibe and PCSK9 inhibitors contribute to an increased survival rate [7, 14, 15]. Currently these patients are still lacking appropriate diagnosis, there is a lack of multidisciplinary approach and there is a high unmet need for management of FH patients in Romania. This prospective study included patients with suspected FH, with the following objectives: (a) to identify FH patients in the North-Eastern part of Romania and to analyze the demographic, clinical and paraclinical data (b) to evaluate the risk of new cardiovascular events at follow-up in these patients stratified by lipid-lowering agents (at baseline and at 12 and 24 months of follow-up, respectively).

## Methods

### Inclusion and exclusion criteria for patients with suspected FH

#### Study design

observational, prospective, 2 years study (October 2017 to October 2019) at three academic medical centers in the North-Eastern part of Romania.

#### Study population

Nine hundred-eighty patients with dyslipidemia were identified between September 2016 and October 2017, and 61 patients met the following ***inclusion criteria:*** subjects with full mental capacity who signed the informed consent form; men and women aged over 18 years. The DLCN score above 3 and the Simon Broome criteria (probable or possible FH) were two important selection tools for the patients with clinical diagnosis of FH. These included the following elements: identification of a family history of hypercholesterolemia or cholesterol deposits in vascular and extravascular tissues; setup of a personal history of early onset of coronary, cerebrovascular and peripheral vascular diseases; clinical observations regarding the presence of either xanthomas, xanthelasma and/or arcus cornealis; biological identification of total cholesterol (TC) > 300 mg/dL, low density lipoprotein cholesterol (LDL-C) > 190 mg/dL without treatment or > 100 mg/dL following treatment with maximum doses of statins (40 mg rosuvastatin, 80 mg atorvastatin, in combination with ezetimibe, as required).

#### Exclusion criteria

subjects lacking discernment or those who refused to sign the informed consent, patients under the age of 18, pregnant and breast-feeding women; subjects with severe physical disabilities, dementia, neoplasms and other causes of secondary hypercholesterolemia (uncontrolled diabetes, nephrotic syndrome, hypothyroidism, drug-induced dyslipidemia) [16].

### Clinical and biological evaluation in FH patients

The follow-up included 3 components: clinical examination data, laboratory investigations and ultrasound parameters.

The study included patients with Dutch Lipid Clinic Network score > 3 for the FH population [9]. The reference values of the DLCN score were: 3–5 points highlighted possible FH, 6–7 points indicated probable FH, while over 8 points indicated definite FH [9]. The other score, namely the Simon Broome score advertised between possible, probable or definitive FH [9]. The patients included in the study were coded with the letter H and the corresponding ID number.

Laboratory analysis included values at baseline, and at 12 and 24 months (stratified by statin alone- atorvastatin 80 mg maximum, rosuvastatin 40 mg, simvastatin 80 mg; combinations between high dose statin and ezetimibe 10 mg and/or fenofibrate 160 mg). Additionally, medical history revealed that certain patients had received antihypertensive medication (those with BP > 140/90 mmHg), or oral antidiabetic medication (those diagnosed with type 2 diabetes), which was allowed throughout the study, according to the specialist doctors’ prescription.

The total cholesterol (TC) mg/dL, low density cholesterol lipoprotein (LDL-C) mg/dL, high density cholesterol lipoprotein (HDL-C) mg/dL, triglycerides (TG) mg/dL, blood glucose (mg/dL), aspartate transaminase (AST) U/L and alanine aminotransferase (ALT) U/L, uric acid (UA) mg/dL, high-sensitivity C-reactive protein (hsCRP) mg/dL were measured by spectrophotometric assay (UV VIS Spectrophotometer-Architect C8000- Abbott Laboratory, USA).

Further explorations for cardiovascular evaluation included:

- an electrocardiogram (ECG) for ischemic changes assessment;
- an ABI measurement with a sphygmomanometer and a portable ultrasonography device, which determines sounds that detect systolic blood pressure in the lower limbs; the reference ABI values were between 0.9 and 1.3.
- echocardiography (Siemens Acuson CV70 Cardiac Vascular Ultrasound Machine) highlighting left ventricular (LV) wall motion abnormalities and ejection fraction values, which are important predictors of left ventricular systolic dysfunction;
- measurement of cIMT (at the levels of carotid bifurcation, internal, external, right and left carotid arteries) by using Siemens Acuson CV70 Cardiac Vascular Ultrasound Machine, B-mode and color Doppler ultrasound (5–10 *MHz*). The average of the cIMT (the average of the six quantified segments) was also recorded. The reference cIMT values were under 0.9 mm [13, 17].

### Evaluation of cardiovascular risk factors and the new cardiovascular events

Treatment goals were defined by the 2019 Guidelines on Dyslipidaemias [9]. ***The cardiovascular risk factors*** were defined according to the European Society of Cardiology (ESC): age and gender (> 50 years for men and > 60 years for women), genetic factors, race and ethnicity, diabetes mellitus (FPG ≥126 mg/dL: fasting was defined as no caloric intake for at least 8 h; 2-h plasma glucose≥200 mg/dL during oral glucose tolerance test: the test should be performed using a glucose load containing the equivalent of 75-g anhydrous glucose dissolved in water; A1C ≥6.5% using a standardized assay, classic symptoms of hyperglycemia or hyperglycemic crisis with a random plasma glucose ≥200 mg/dL), obesity (BMI exceeding 30 kg/m^2^, waist circumference in Caucasian females > 88 cm and in males > 102 cm), physical inactivity (under 30–60 min activity on most days), smoking (active or passive or without exposure to tobacco in any form) and high blood pressure (BP> 140/90 mmHg) [9, 15, 18, 19].

***Atherosclerotic cardiovascular disease (ASCVD)*** was defined as a history of one of the following diseases as identified in the medical records: coronary heart disease (CHD) with the following particularities: acute coronary syndrome, myocardial infarction (MI), stable angina, coronary revascularization, ischemic stroke, or transient ischemic attack and peripheral artery disease (PAD) [20].

### Statistical analysis

The data of the patients with clinical diagnosis of FH were introduced into a database and processed through the statistical functions of the SPSS version 20.0 system. One-sample Kolmogorov-Smirnov for normal distribution tests were performed, with the data being calculated as: mean and standard deviation (SD) for normal distribution variables, percent for categorical variables by using a frequency test, median and interquartile range (IQR) for continuous variables with asymmetrical distribution. Bivariate correlation analysis was achieved between the scale variables, using the Pearson correlation coefficient. To analyze the associations between ordinary and/or nominal variables with specific variables, specific association coefficients were used (Cramer’s, Phi, contingency coefficient). Comparative analyses between the pathological, family history, clinical and paraclinical history according to gender were performed for the values ​​that did not meet the criteria of normal homogeneity, whereas normal distribution was performed for nonparametric tests: Mann-Withney U sample, Wilcoxon Signed-rank, Kruskall-Wallis H test, Friedman test. They were also employed for the categorical variables Chi Square (χ^2^) and Fisher Exact Test. Survival free of ASCVD, defined as cardiovascular events (CHD, stroke, PAD) during follow-up, was estimated using the Kaplan-Meier method. The duration of follow-up was calculated from the date of inclusion in the study to the date of the cardiovascular events. Multiple logistic regression analysis was applied to identify the independent factors for cardiovascular events. The *P* value < 0.05 was considered statistically significant.

## Results

### Baseline data of patients with suspected FH in the North-Eastern part of Romania

**Fig. 1:**
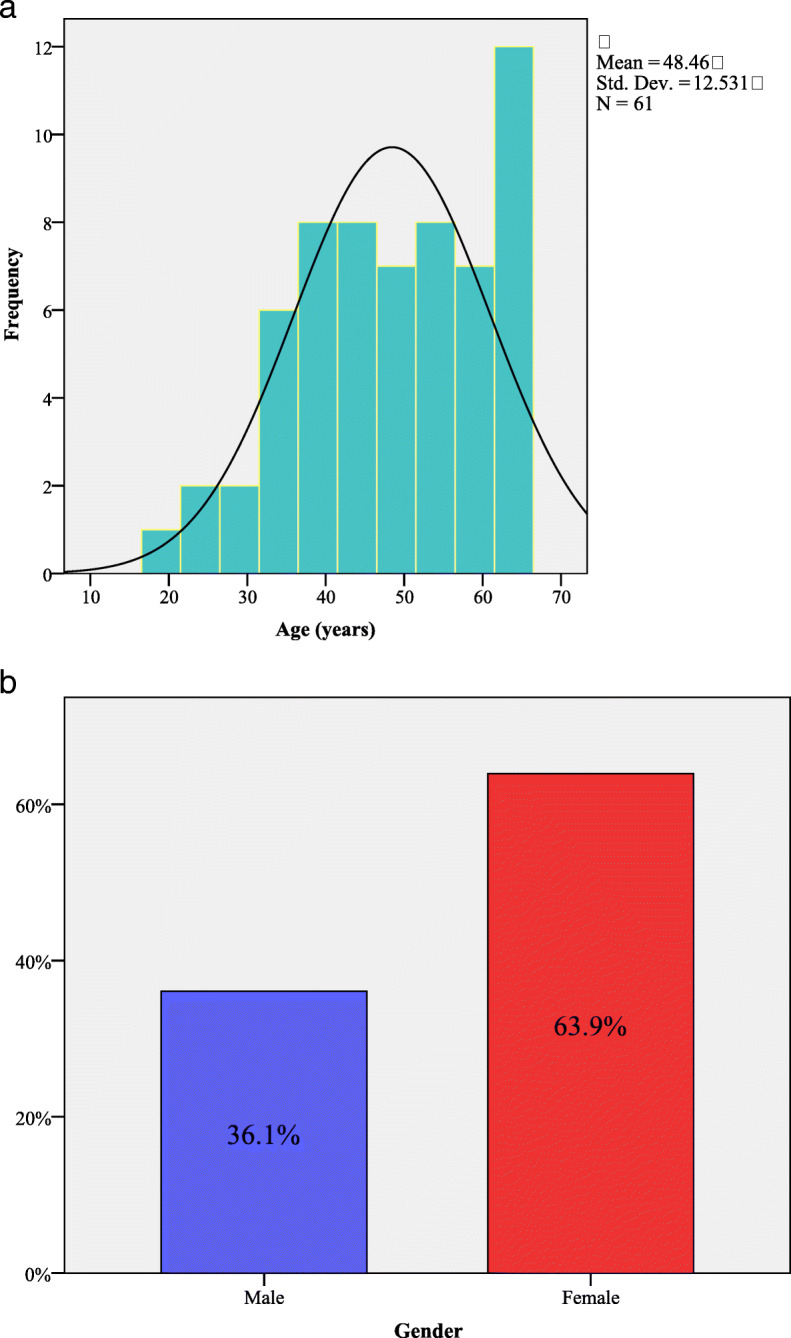
The frequency of FH patients according to **a.** age and **b.** gender

**Fig. 2:**
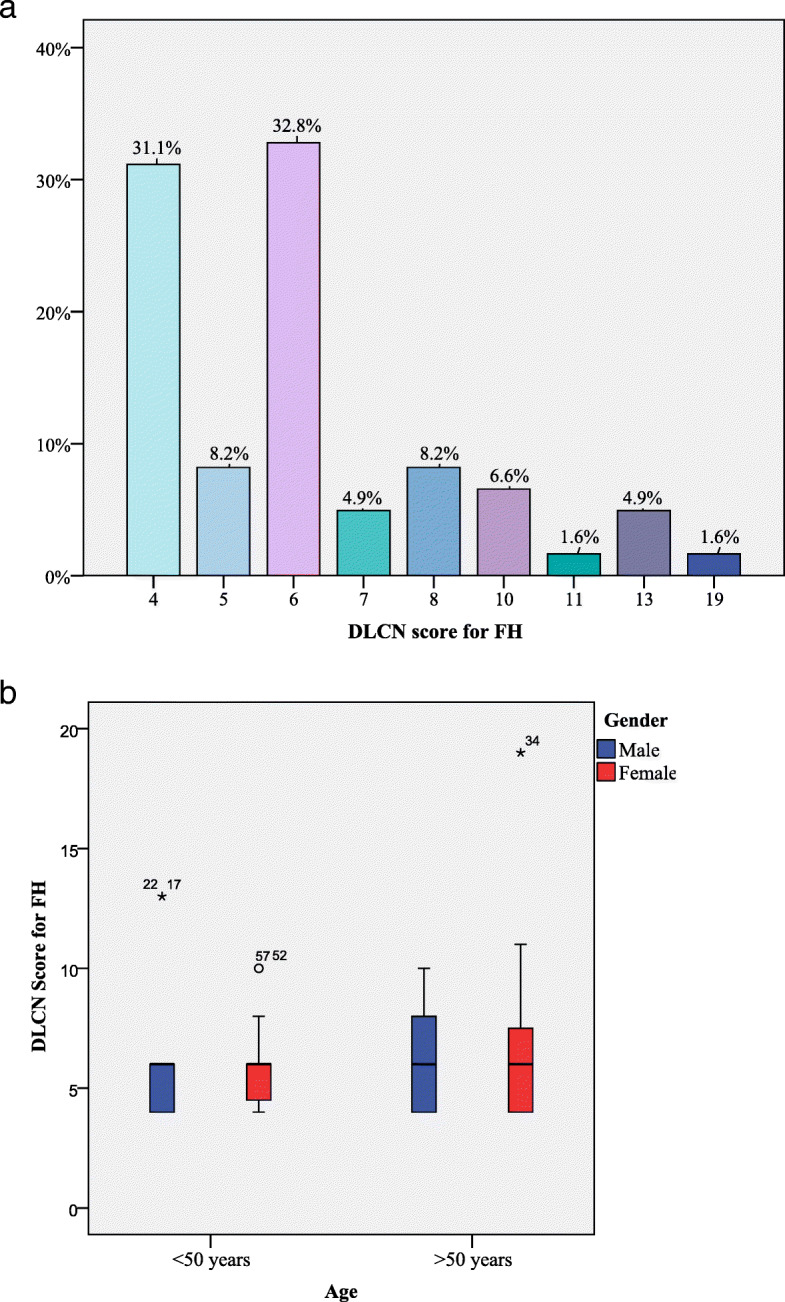
The frequency of the DLCN score and the distribution of the score by **a**. age and **b**. gender

The study group included 61 patients (6.2% of all patients examined), with a mean age of 48.5 ±12.5 years, all subjects being Caucasian (Fig. 1a. and Fig. 1b.), with a higher number of women compared to men (63.9% versus 36%). The laboratory results were: TC 315 ± 56 mg/dL, LDL-C 254.2 ± 53 mg/dL, HDL-C 45.8 ± 18 mg/dL, TG 174.4 ± 92 mg/dL (for all patients), whereas the lipid profile did not differ according to gender (Table 1). Moreover, 36.1% of the patients had ASCVD history. Table 1 presents the demographic and clinical data of the patients with clinical diagnosis of FH, as well as the main cardiovascular risk factors for male and female patients. Furthermore, uric acid and smoker status (active or passive) displayed different values according to gender. At baseline, all the patients in the study had lipid-lowering therapies (about 1 year of treatment until inclusion in the study) the most frequent being the treatment with statin monotherapy 36.1%, followed by the associations between statin and ezetimibe, statin and fenofibrate, and the triple combination between them respectively. 3% of the study patients reported adverse effects to statin such as myalgia and headache (Table 1). The FH status of the enrolled subjects was assessed by calculating the DLCN score, with reference values between 4 and 19. ***Possible FH*** was identified with a score of 4 in 31.2% (*n* = 19 patients), a score of 5 in 8.2% (*n* = 5 patients). ***Probable FH*** was identified with a score of 6 in 32.8% (*n*=20 patients), a score of 7 in 4.9% (*n*=3 patients), a score of 8 in 8.2% (*n*=5 patients). ***Definite FH*** was identified with a score of 10 in 6.6% (*n*=4 patients), a score of 11 in 1.6% (*n*=1 patient), a score of 13 in 4.9% (*n* = 3 patients) and a score of 19 in 1.6% (*n*=1 patient) (Fig. 2a. and Fig. 2b.). Moreover, the DLCN score showed no variations between patients, either according to gender (U = 432.5, z = − 0.05, *P* = 0.95) or the age (U = 494, z = − 0.45, *P* = 0.65). According to the Simon Broome score, patients were classified as possible FH (*n* = 47, 77%) and probable FH (*n* = 14, 23%). Both ischemic changes on the ECG and LV wall motion abnormalities following echocardiography were identified in the 24 patients suspected of having FH,, while in the other patients (*n*=37) these pathological aspects were not observed (χ^2^(1) = 61, *P* = 0.001). TC values were correlated with increased cIMT values (r = + 0.37, *P* = 0.03), with low values of ejection fraction (EF) (r = − 0.43, *P* = 0.001) and with low ABI levels (r = − 0.64, *P* = 0.001). The significantly increased LDL-C values were positively correlated with high values of cIMT (r = + 0.39, *P* = 0.002) and negatively correlated with low EF values (r = − 0.42, *P* = 0.001), and low ABI values (*P* = 0.001). The significantly increased TG concentrations were positively correlated with high values of cIMT (r = + 3.30, *P* = 0.02), while low HDL-C did not correlate with any of the parameters. In addition, nontraditional cardiovascular risk factors represented by hsCRP and uric acid were correlated as follows: high values of hsCRP were positively and significantly correlated with high concentrations of TC (r = + 0.45, *P* = 0.001) and LDL-C (r = + 0.47, *P*=0.001), and the increased values of uric acid were positively correlated with the higher TG (r = + 0.29, *P*=0.02) values.

**Table 1:** Baseline characteristics of patients suspected for FH

Characteristics	Patients suspected for FH			
	Total	Male	Female	***P***
N	61	22	39	0.001
Age - yo (mean±SD)	48.4 ± 12.5	46.1 ± 1	49.7 ±1	0.4
Smoker *n* (%)	18 (29.5%)	12 (54.5%)	6 (15.4%)	0.001*
High blood pressure n (%)	31 (50.8%)	13 (59.1%)	18 (46.2%)	0.3
CHD history *n* (%)	28 (21.3%)	6 (27.3%)	22 (17.9%)	0.5
PAD history *n* (%)	10 (14.8%)	3 (13.6%)	7 (15.4%)	0.5
CHD+PAD history *n* (%)	11 (23%)	5 (22.7%)	6 (23.1%)	0.5
Obesity n (%)	36.1%	8 (36.4%)	14 (35.9%)	0.9
Type 2 diabetes *n* (%)	8 (13.1%)	4 (18.2%)	4 (10.3%)	0.4
Physical inactivity *n* (%)	30 (49.2%)	14 (63.6%)	16 (41%)	0.09
TC mg/dL (median±IQR)	315 ± 56	313.5 ± 41	315 ±60	0.7
LDL-C mg/dL (mean±SD)	254.2 ± 53	257 ±63	252.5± 47	0.8
HDL-C mg/dL (median± QR)	45.8 ±18	44 ±19	46 ±18	0.6
TG mg/dL (mean±SD)	174.4 ± 92	163.7± 90	180.5± 94	0.6
Uric acid mg/dL (mean±SD)	5.79 ± 1.22	6.2 ±1.1	5.5 ±1.2	0.02*
hsCRP mg/L (mean±SD)	5.85 ± 2.29	6.3 ±2.5	5.9± 2	0.5
ECG changes *n* (%)	25 (41%)	11 (50)	14 (35.9)	0.3
LV wall motion abnormalities *n* (%)	25 (41%)	11 (50)	14 (35.9)	0.1
ABI (mean±SD)	0.96±0.93	0.84±0.09	1.01±1.4	0.8
cIMT mm (mean±SD)	0.95±0.33	1.02±0.34	0.9±0.32	0.2
Lipid-Lowering Agents				0.3
Statin *n* (%)	22 (36.1%)	7(39.8%)	15(38.5%)	
Statin + ezetimibe *n* (%)	18 (29.5%)	11 (50%)	7 (17.9%)	
Statin + fenofibrate *n* (%)	8 (13.1%)	3 (13.6%)	5 (12.8%)	
Statin + fenofibrate + ezetimibe n (%)	13 (21.3%)	1 (4.5%)	12 (30.8%)	
Adverse effects *n* (%)	3%	1%	2%	

Legend: *CHD* coronary heart disease, *PAD* Peripheral arterial disease, *TC* Total cholesterol, *LDL-C* low density cholesterol lipoprotein, *HDL-C* high density cholesterol lipoprotein, *TG* triglycerides, *hsCRP* high-sensitivity C-reactive protein, *ECG* electrocardiogram, *LV-left* ventricular, *ABI* ankle-brachial index, *cIMT* carotid intima-media thickness

*P< 0.05

### ASCVD in patients with suspected FH follow-up based on lipid lowering drugs

**Fig. 3:**
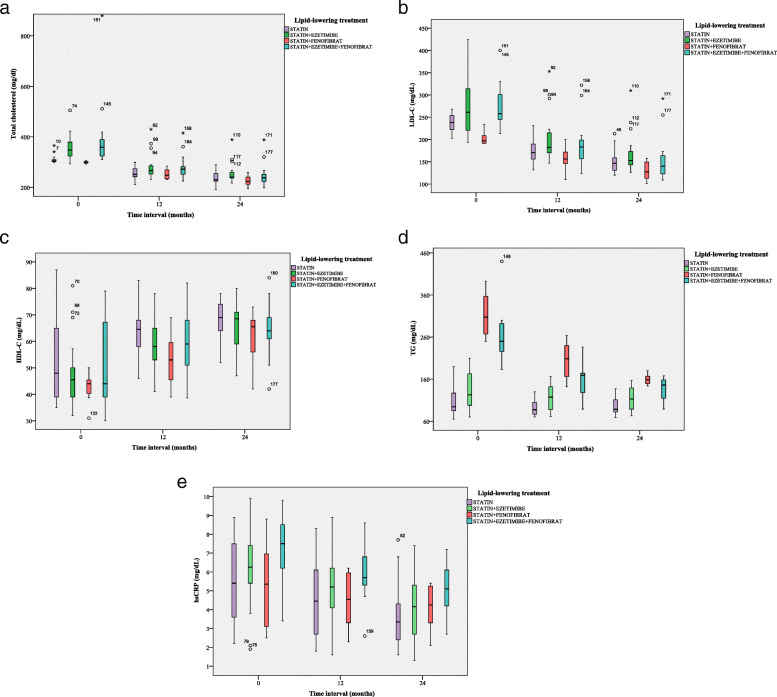
Lipid profile (**a**. total cholesterol; **b**. LDL-C; **c**.HDL-C; **d**.TG; e.hsCRP) based on the lipid-lowering treatment and the effect of time interval

**Fig. 4:**
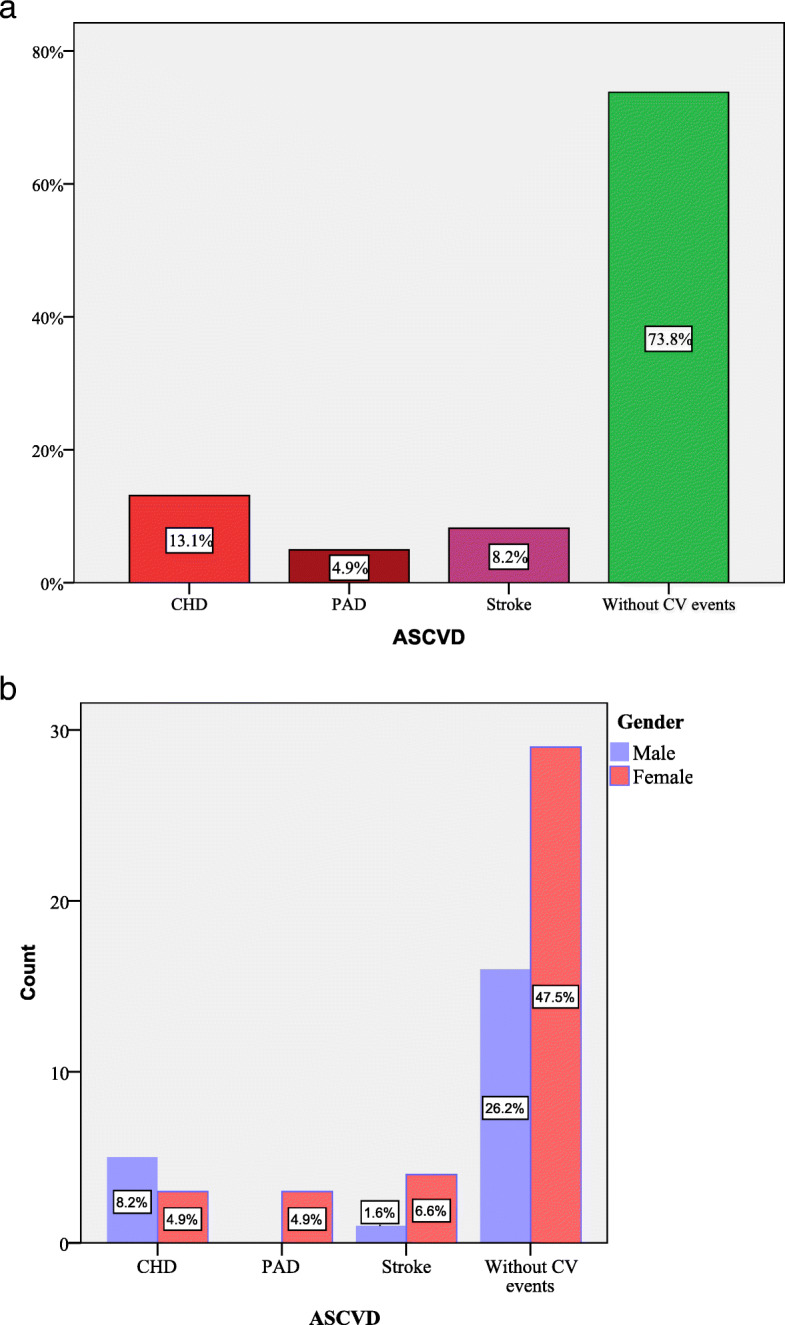
The frequency of the new ASCVD **a.** in population with clinical diagnosis of FH **b.** in this population by gender

**Fig. 5:**
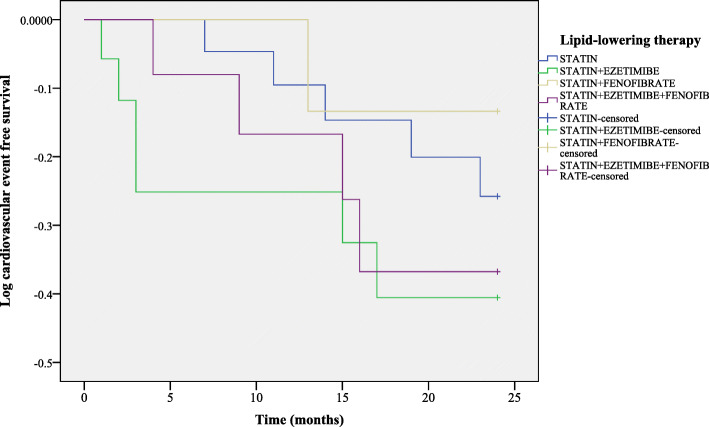
Kaplan Meier for ASCVD depending on the lipid-lowering therapy and time interval for the occurrence of new cardiovascular events

Intensive lipid-lowering therapy administered for 12 months and 24 months respectively, compared to baseline, either statin alone and statin in association with fenofibrate, were found to decrease the TC levels (Fig. 3a). In addition, a significant reduction of LDL-C concentrations was observed at 12 months after the enrollment in the study, for the patients with maximum treatment dose (statin in association with fenofibrate) compared to baseline, but with a minimum decrease, after 24 months (Table 2 and Fig. 3b). Furthermore, at both 12 months and 24 months follow-ups, the most efficient treatment to improve HDL-C values and to decrease TG and hsCRP levels was statin alone (Fig. 3c-e and Table 2). At the same time, both ABI and cIMT levels recorded significant differences between the groups of patients receiving lipid-lowering agents after 24 months of follow-up (Table 2). On the other hand, lipid-lowering therapy affected significantly neither the blood glucose levels, nor transaminases levels (*P*> 0.05) (Table 2). In this study, 26.2% (*n*=16 patients) of the population with clinical diagnosis of FH displayed new cardiovascular events during the follow-up, as follows: CHD in 13.1% of 61 enrolled patients (*n*=8 patients), stroke in 4.9% of 61 enrolled patients (*n*=3 patients) and PAD in 8.2% of 61 enrolled patients (*n*=5 patients) (Fig. 4a). More female had new cardiovascular events represented by PAD (4.9% of 61 enrolled patients, *n*=3 patients) and stroke (6.6% of 61 enrolled patients, *n*=4 patients), as compared to men, in which CHD occurred more frequently (8.2% of 61 enrolled patients, *n*= 5 patients) (Fig. 4b). For the subjects who received statin associated with fenofibrate (23 months), respectively high-dose of statin alone (22 months), the time-interval for ASCVD (composite endpoint) occurrence was not significantly postponed, as compared to patients receiving the 3 lipid-lowering drugs association (20 months) or statin associated with ezetimibe (18 months) (log rank χ^2^ = 1.7, *P* = 0.6) (Fig. 5). Furthermore, following the multiple logistic regression, only LDL-C over 190 mg/dL, and hsCRP > 5 mg/L were predictors of cardiovascular events in patients with clinical diagnosis of FH (Table 3).

**Table 2:** Paraclinical characteristics of patients with clinical diagnosis of FH on the follow-up

Characteristics	Patients with clinical diagnosis of FH			
	Baseline	12 months	24 months	***P***
TC mg/dL (median ± IQR)	315 ± 56	258 ± 37	237 ± 33	0.001*
LDL-C mg/dL (mean ±SD)	254.2 ± 53	185± 46	156 ± 40	0.001*
HDL-C mg/dL (median± IQR)	45.8 ±18	59 ± 15	68 ± 11	0.001*
TG mg/dL (mean ± SD)	174.4 ± 92	130± 49	119 ± 32	0.001*
Uric acid mg/dL (mean ± SD)	5.8 ± 1.22	5.3 ± 0.9	5.1 ± 1	0.001*
hsCRP mg/L (mean ± SD)	5.9 ± 2.29	5 ± 1.9	4.2 ± 1.7	0.001*
Glucose (median ± IQR)	97 ± 18	100 ± 15	97 ± 23	0.06
AST (median ± IQR)	25 ± 14	28 ± 15	28 ± 31	0.07
ALT (median ± IQR)	28 ± 20	33 ± 15	32 ± 15	0.2
ABI (mean ± SD)	0.96 ± 0.9	0.92 ± 0.6	0.91 ± 0.08	0.001*
cIMT mm (mean ± SD)	0.95 ± 0.3	0.91 ± 0.3	0.85 ± 0.3	0.001*

Legend: *TC* Total cholesterol, *LDL-C* low density cholesterol lipoprotein, *HDL-C* high density cholesterol lipoprotein, *TG* triglycerides, *hsCRP* high-sensitivity C-reactive protein, *AST* aspartate transaminase, *ALT* alanine aminotransferase, *ABI* ankle-brachial index, *cIMT* carotid intima-media thickness

*P< 0.05

**Table 3:** Independent factors for cardiovascular events in patients suspected for FH

Variables	OR	95% C.I. for OR		*P*
		Lower	Upper	
cIMT baseline	4.2	0.1	178	0.5
ABI baseline	0.2	0.1	87	0.4
LDL-C at baseline	1.1	1.01	1.2	0.007*
hsCRP at baseline	1.7	1.03	2.81	0.04*
Lipid-lowering drugs				
Statin+Ezetimibe+Fenofibrate	ref	ref	ref	0.1
Statin	206	2.7	1583	0.02*
Statin+Ezetimibe	17.5	0.4	693	0.1
Statine+Fenofibrate	992	2.2	4523	0.03*

Legend: *LDL-C* low density cholesterol lipoprotein, *hsCRP* high-sensitivity C-reactive protein, *cIMT* carotid intima-media thickness, *OR* odd ratio

*P< 0.05

## Discussion

This is the first epidemiological study aiming at identifying the population with FH in Romania’s NE region, which includes a population of approximately 3.979.978 inhabitants This study represents a milestone in the identification of cases with familial dyslipidemia, in order to make the differential diagnosis between FH and polygenic dyslipidemia. In Romania, there is no FH registry. The findings of the current study highlight the necessity for creating a database. This, in turn, will support the implementation of a national policy for early screening through genetic tests, in order to find out the approximate number, the phenotype and genotype of FH patients in the different regions of Romania, and it can create the basis for a targeted management with monoclonal antibodies resulting in the reduction of new cardiovascular events; finally, international collaborations will lead to a better understanding of this pathology. In the aforementioned study, 980 patients with dyslipidemia from the North-Eastern part of Romania were evaluated, measuring the DLCN and Simon Broome (SM) scores, a number of 61 patients with DLCN score above 3 and possible/probable FH at SM score being included. The demographic data identified more female patients than male patients, indicating that women address the primary health care units earlier. The laboratory tests acknowledged significantly higher values of lipid profile by gender stratification, except for TG levels (the female patients had greater TG compared to male patients: 180.5 ± 94 mg/dL vs 163.7 ± 90 mg/dL). In contrast to this study, Casula et al., in a study of FH patients from Italy, identified no significant differences between genders, the means levels of lipid panel being close to those recorded by the subjects included in this study [21]. Hypertension was the main cardiovascular risk factor for both sexes, followed by sedentary lifestyle and obesity for the female group and active or passive smoking for the male group. As in the studies conducted by Averna et al. and Yudi et al., this study revealed the similar results regarding the DLCN score between 3 and 6 (possible FH or probable FH), but different results for the DLCN score above 8 (definite FH) - they have a higher number of patients with DLCN> 8 [22]. Patients with suspected FH were identified with ischemic changes on ECG and LV wall motion abnormalities (LV hypokinesia or akinesia) on echocardiography, as in the case of Asian FH patients included in the study conducted by Song et al. [23]. hsCRP is a nontraditional marker of cardiovascular risk, which occurs in all stages of atherogenesis and correlates with different cardiovascular pathologies [24]. Likewise, in a cross-sectional study, Eltoft et al. pointed out that hsCRP was associated with the identification of atherosclerotic plaques, but without influencing the formation of new plaque or its progression [25], equivalent findings being also observed in the above-mentioned study. The ankle-brachial index (ABI) had significantly lower values in men compared to women, with male patients frequently displaying subclinical atherosclerosis. Moreover, this specific group of patients needs screening for the early identification of PAD, even if they are asymptomatic, an approach also supported by Pereira et al. [26]. In this study, the marker of subclinical atherosclerosis represented by cIMT correlated poorly with dyslipidemia (with increased TC, LDL-C and TG levels, but not with decreased HDL-C concentration), an idea supported by Khan et al. (mean cIMT was significantly correlated with LDL-C) [13]. In contrast to the results described above, in a meta-analysis that included 51 studies and 4057 FH patients, Masoura et al. showed that mean cIMT correlates neither with LDL-C levels, nor with TG values in the FH population [12]. Also, in the ASAP study, Smilde et al. found the same trend regarding the baseline mean of cIMT grouped by gender [27]. Likewise, in an observational study, Elis et al. found the same frequency of lipid-lowering agents, with a higher administration of statin in monotherapy compared the combination of these drugs [28]. Furthermore, in this study, the adverse effects (myalgia and headache) affected a small number of patients (3%), similar to the results reported by Elis et al. (none of the FH patients had severe adverse reactions such as hepatic impairment or rhabdomyolysis) [28]. Even though this study indicates that the lipid-lowering therapy did not cause significant changes on glycemic levels or transaminases concentrations, the glycemic control and liver function tests should be performed for safety reasons [9]. This research indicates that all lipid-lowering drugs have contributed to the postponement of cardiovascular events, without significant differences between them. The administration of the lipid-lowering agents during the follow-up resulted in decreased TC and LDL-C levels and elevated HDL-C levels, yet without reaching the goals established by the European Guide of Dyslipidemias. In order to achieve the target objectives, it is necessary to discuss the introduction of monoclonal antibodies in lipid-lowering medication combinations, a conclusion also supported by Cesaro et al. [29]. The PCSK9 inhibitors had a good adherence (a good safety profile and a good efficacity) [2, 30]. The PCSK9 inhibitors represent a valuable therapy in patients with FH, with a significant reduction in LDL-C values, their bi-weekly or monthly administration contributing to an increased quality of life [29]. Yet, because of the low economic level and moderate addressability of health care, this medication is difficult to implement as a national public health program. Similarly, Khan et al. found that increased hsCRP and LDL-C levels (caused by prolonged exposure of vessels to high cholesterol levels) were associated with a significantly increased risk of CHD [13]. Thus, several authors reached conclusions that are similar to those presented in the study conducted in this region of Romania, as indicated in Table 4.

**Table 4:** Characteristics of different studies for cardiovascular outcomes

Author	Study type	FH Population	Country	Outcomes	Results
Sivapalaratnam et al. 2010 [17]	Observational study	40 FH patients	Netherlands Amsterdam	the effectiveness of statins on reducing the arterial wall thicknesses (cIMT)	• Pre-treatment total cholesterol levels of FH patients were on average 9.3±2.0 mmol/L. • Pre-treatment, total cholesterol levels of FH patients were on average 9.3±2.0 mmol/L, whereas treated HF patients had LDL-C levels between 8±1.5 mmol/L and total cholesterol levels between 5.8±1.6. • Long-term statin treatment reduced cIMT values in severe FH patients.
Pérez de Isla et al. 2017 [20]	multicentre, nationwide, long-term prospective cohort study SAFEHEART	2404 adult patients with FH (molecularly defined population)	Spain Madrid	defining the key risk factors for predicting incident ASCVD	• During the follow-up of the study (5.5 years), 122 patients (5.1%) suffered fatal and nonfatal incident ASCVD, respectively. • Age, male sex, history of previous ASCVD, high blood pressure, increased body mass index, active smoking, and low-density lipoprotein cholesterol and Lp(a) levels were independent predictors of incident ASCVD.
Junyent et al. 2008 [31]	Case-control study	146 FH patients carrying null alleles (*n*=48), defective-receptor alleles (*n*=62), undetermined-function alleles (*n*=25), or APOB defects (*n*=11)	Spain, Barcelona	molecularly defined heterozygous FH in comparison with matched control subjects	• 23 patients had coronary heart disease (CHD). • The frequency of both tendon xanthomas and CHD was 2-fold higher than in the control group. • All femoral intima-media thickness (IMT) measurements were increased in FH patients versus patients in the control group (*P*=0.001). • On multivariate analysis, the mean of IMT (a measure of early atherosclerosis) was independently associated with age, LDL-C, sex, and systolic blood pressure.
Perak et al. 2016 [32]	6 large epidemiological cohorts	3850 (5.6%) had the FH phenotype by the primary definition (LDL-C levels ≥190 mg/dL and < 130 mg/dL)	USA, Chicago	coronary heart disease (CHD) total atherosclerotic cardiovascular disease (ASCVD) risks	• After covariate adjustment, the FH phenotype was associated with high 30-year CHD risk (HR= 5, 95% CI: 1.1–21.7, *P*< 0.005). • CHD risk was increased by 10 to 20 years in men and 20 to 30 years in women. • Total ASCVD risk was elevated (HR=4.1, 95% CI: 1.2–13.4, *P*< 0.005) • FH phenotype definitions which included family history, LDL-C thresholds, or alternative lipid fractions, decreased the FH phenotype prevalence to 0.2–0.4%, without affecting the CHD risk (HR=8.0; 95% CI:1.0–61.6, *P*< 0.05).
Pereira et al. 2015 [33]	observational cross-sectional study	202 patients with heterozygous FH	Brazil, São Paulo	association of peripheral artery disease PAD with other manifestations of cardiovascular disease (CVD)	• The mean age was 51 ± 14 years, 35% men and total cholesterol levels were 342 ± 86 mg/dL. • The prevalence of PAD and previous CVD were 17 and 28.2%. • On multivariate analysis, CVD was independently associated with the diagnosis of PAD (OR = 2.50; 95% CI: 1.004–6.230; *P* = 0.049).
Nanchen et al. 2016 [34]	a multicentre, prospective cohort study	945 patients with clinical diagnosis of FH	Switzerland Lausanne	the occurrence of CV events during the first year after hospitalization for ACS	• The prevalence of FH was 5.5% with the Simon Broome definition, and 1.6% with the Dutch Lipid Clinic score. • After multivariable adjustment including age, the risk was greater in patients with FH than in those without, with an adjusted HR =2.73 (95% CI: 1.46–5.11; *P*=0.002) for the Simon Broome definition and an adjusted HR =3.53 (95% CI: 1.26–9.94; *P*=0.017) for the Dutch Lipid Clinic definition. • Patients with FH and ACS have a > 2-fold adjusted risk of coronary event recurrence within the first year after discharge than patients without FH despite the widespread use of high-intensity statins.
Besseling et al. 2016 [35]	retrospective cohort study	1559 He FH patients	Netherlands Amsterdam	the relative risk reduction for CAD and for mortality by using statins	• In heterozygous (He) FH patients, the moderate - to high intensity statin therapy reduced the risk for CAD and the mortality by 44%.
Brunham et al. 2016 [36]	longitudinal observational study	339 patients with clinically diagnosed HeFH	Vancouver, Canada	characterizing the clinical features, the treatment patterns and CV outcomes	• The overall CV event rate was 33.5/1000 person -years. • Among patients that had a CV event during the follow up, 59% experienced a recurrent event within 5 years. • After using the lipid-lowering therapies, ≥50% reduction in LDL-C was achieved in 34.5% of the patients, and an LDL-C ≤2 mmol/L in 8.3%. Despite a majority of patients receiving lipid lowering therapy, few patients reached the lipid targets.
Emanuelsson et al. 2018 [37]	prospective cohort study of the general population	7109 were diagnosed with FH	Denmark Copenhagen	establishing the PAD risk and the relationship between ABI and myocardial infarction	• In multivariable adjusted ORs, PAD were 1.84 (95% CI: 1.70–2.00, *P*=0.001) in those with possible FH and 1.36 (95% CI: 1.00–1.84, *P*=0.001) in individuals with probable/definite FH compared with patients with unlikely FH. • The myocardial infarction was 4.60 (95% CI: 2.36–8.97, *P*=0.001) in those with possible/probable/definite FH and ABI< 0.9, compared with individuals with unlikely FH and ABI > 0.9.
Faggiano et al. 2018 [38]	observational multicentre nationwide survey	368 with DLCN score> 3	Italy	evaluating the prevalence of potential FH and the therapeutic approaches among patients with established coronary artery disease (CAD) or PAD	• The prevalence of potential FH was 3.7%. • Men represented 83.7% of the sample; the mean age was 65.9±10.6 years. • The most common clinical presentation was new ACS, with or without percutaneous myocardial revascularization (52.5%), followed by stable CAD on medical therapy (26.5%); isolated lower extremity PAD was the least common presentation (3.1%). • Definite FH (DLCN score> 8) had the highest percentages of patients after an ACS (75% vs 52.5% in the whole study population). • At discharge, most patients were on high intensity statin therapy, they still had higher LDL-C levels, but without reaching the guideline’s goals.
Petrov et al. 2018 [39]	observational study	196 patients with FH diagnosis	Bulgaria, Sofia	the examination of the clinical characteristics and the management of FH over a 12-month period	• The mean age was 54.4 years, 64.1% of subjects were males. • Out of 196 patients the following number of patients met the criteria for FH diagnosis: 27 for definite FH, 94 for probable FH and 75 for possible FH. • At baseline, the mean CV risk classification was 26.8% for CV high-risk and 73.2% for CV very high-risk. • At enrolment, the LDL-C levels were 5.6 mmol/L and 4.1 mmol/L at the last observation visit (12 months). • Most subjects (*n*=219) received statins, but without reaching the ESC/EAS defined LDL-C targets. Intensive statin treatment (atorvastatin 40–80 mg/daily and rosuvastatin 20–40 mg/daily) was used in 38.6% of the patients and 10% of the subjects received combined therapy (statin plus ezetimibe or other LLT). One subject was statin intolerant (ezetimibe therapy).
Al-Rasadi et al. 2018 [40]	multicenter cohort (Gulf COAST cohort)	1030 patients with clinical FH	Arabian Gulf	assessing the prevalence of FH, its management, and impact on ASCVD	• At admission, the proportion of “probable/definite”, “possible”, and “unlikely” FH in ACS patients was 3.7% (*n* =119), 28% (*n*=911), and 68% (*n*=2194). • The “probable/definite” FH group had a greater prevalence of early coronary disease (38% vs 8.8%; *P*< 0.001), and previous statin use (87% vs 57%; *P* < 0.001) compared with the “unlikely” FH group. • After 1 year of follow-up, the “probable/definite” FH cohort had worse lipid control (13% vs 23%; *P*< 001) and presented a greater association with the composite ASCVD endpoint when compared with the “unlikely” FH group (OR=1.85; 95% CI: 1.01–3.38; *P*=0.047).
Teramoto et al. 2018 [41]	retrospective observational study	3.495 FH patients 193 patients were existing diagnosis of FH (FH-D) and 3339 patients were suspected FH (FH-S)	Japan	evaluation of the epidemiology and the treatment patterns associated with lipid-modifying therapies	• The mean LDL-C levels were 147.6 mg/dL for patients with FH-S and 119.2 mg/dL for FH-D. • 55.5% of the patients were treated with lipid-lowering therapy: high-intensity statins in 19.2% of the FH-D patients and 2.3% of the FH-S patients. • Among the FH-D and FH-S statin treated patients, 69.3 and 89.7% respectively remained on monotherapy even when their LDL-C was ≥100 mg/dL. • The therapy and management of LDL-C in Japanese FH patients remain suboptimal.
Lalić et al. 2018 [42]	retrospective observational study	302 FH patients treated continuously with statins during 3 years	Serbia Belgrade	analyzing the effect of statin therapy on attainment of LDL-C treatment targets and appearance of new ASCVD and diabetes	• The high intensity statin was prescribed in 17.9% of the cases. • LDL-C levels were significantly lower after 3 years of statin treatment (3.61 ± 1.19 mmol/l) vs. baseline (4.51 ± 1.69 mmol/l; *P* < 0.01) • 6.9% of FH patients reached the recommended ≥50% LDL-C reduction and 16.2% attained the LDL-C < 2.6 mmol/l target. • 9.6% of FH patients developed new ASCVD, with lower HDL-C levels after 3 years of statin treatment, as compared to those who remained free of ASCVD.

## Study strengths and limitations

This is the first epidemiological study conducted in order to identify the population with FH in Romania’s NE region, as it is absolutely necessary to create a database, since currently no active FH registry is available. The region is homogenous, with regard to genetic elements, culture, eating habits, and socio-economic status and is served by several county hospitals and one referral university and research center.

Nevertheless, this study had significant limitations. The patients were followed-up for only 2 years. Secondly, the small number of patients included in the study might suggest that FH is a relatively rare genetic pathology, their selection being made according to the scores provided by the 2019 Guidelines on Dyslipidemias [9]. Thirdly, the current study enrolled subjects from the North-Eastern area of Romania, and current group of subjects did not significantly mirror the entire Romanian population with ASCVD. Moreover, for most enrolled patients there were no values for cIMT, ABI and lipid profile prior to their inclusion in the study.

## Conclusion

The patients with clinical diagnosis of FH included in the study were young patients, mainly women, whose lipid profile did not differ according to gender. Hypertension was the main cardiovascular risk factor, followed by a sedentary lifestyle and obesity for female patients, and by an active smoking status for male patients. Moreover, the DLCN score did not differ according to gender or age, and a significant number (36.1%) of patients had a history of ASCVD. At the end of the study, it was found that lipid-lowering drugs decreased LDL-C, hsCRP and cIMT levels, and increased ABI and HDL-C values, yet without reaching the goals established by the European Guide of Dyslipidemias. The most common new ASCVD event was CHD, followed by stroke and PAD. All lipid-lowering drugs delayed the new cardiovascular events, without significant differences between them. Even though this was the first observational study in the North-Eastern part of Romania, further molecular genetics studies are needed to confirm the FH cases and to evaluate the opportunity for introducing monoclonal antibodies (alirocumab, evolocumab) as a viable and efficient therapy option, despite the socio-economic barriers.

## Data Availability

The data and material of this study are available from the author (L.F.) on reasonable request.

## Abbreviations

FH: Familial hypercholesterolemia
DLCN: Dutch Lipid Clinic Network score
ASCVD: Atherosclerotic cardiovascular disease
CVD: Cardiovascular diseases
CHD: Coronary heart disease
PAD: Peripheral arterial disease
cIMT: Carotid intima-media thickness
ABI: Ankle-brachial index
ECG: Electrocardiogram
LV: Left ventricular
EF: Ejection fraction
TC: Total cholesterol
LDL-C: Low density cholesterol lipoprotein
HDL-C: High density cholesterol lipoprotein
TG: Triglycerides
hsCRP: High-sensitivity C-reactive protein
CI: Confidence interval
HR: Hazard ratio
IQR: Interquartile range
SD: Standard deviation
